# Congenital orbital teratoma: a case report and challenges of its management in a resource limited setting

**Published:** 2012-05-04

**Authors:** Olufunmilola Abimbola Ogun, Gabriel Olabiyi Ogun, Biobele Jotham Brown, Adedamola Lameed Mosuro, Adeyinka Olusola Ashaye

**Affiliations:** 1 Departments of Ophthalmology, University of Ibadan, University College Hospital, Ibadan, Nigeria; 2 Departments of Pathology, University of Ibadan, University College Hospital, Ibadan, Nigeria; 3 Departments of Paediatrics, University of Ibadan, University College Hospital, Ibadan, Nigeria

**Keywords:** Congenital, orbit, immature, teratoma

## Abstract

We report a case of congenital immature teratoma of the orbit in a female neonate who presented on the second day of life. She was successfully managed by modified exenteration. The patient was lost to follow-up intermittently over a 24-month period without recurrence of the tumour. However the patient could not be traced again after 24 months of follow up. This happened despite concerted efforts to educate the parents. The possible implications of this and other social factors, in a challenging and resource limited setting, on the prognosis of the disease and cosmetic outcome are considered.

## Introduction

Congenital orbital teratomas are rare tumours that usually present with progressive unilateral proptosis in a neonate [1]. The tumour may grow rapidly, causing destructive proptosis and exposure keratopathy within days, with resultant poor prognosis for vision or conservation of the globe. Typically, it consists of tissues derived from all three germinal layers [1–10]. Orbital teratomas have been known to recur and may very rarely be associated with a secondary malignancy [2]. However, most congenital orbital teratomas have been shown to be largely benign in biologic behaviour and are now being managed more conservatively with good cosmetic results and a better prognosis for vision [3–5]. We present a case of congenital orbital teratoma from South-Western, Nigeria and with a discussion on the challenges faced during the course of management and follow up.

## Patient and case report

**Figure 1:**
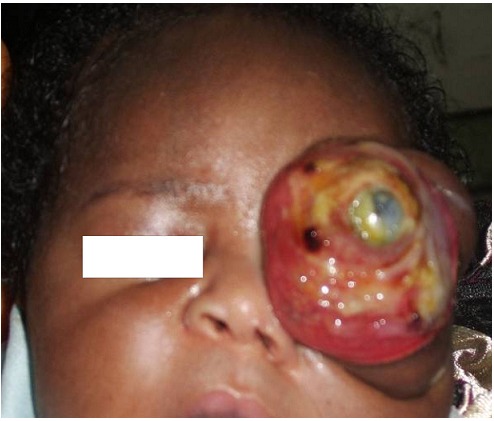
Massive proptosis with exposure keratopathy on 5th day of life

**Figure 2:**
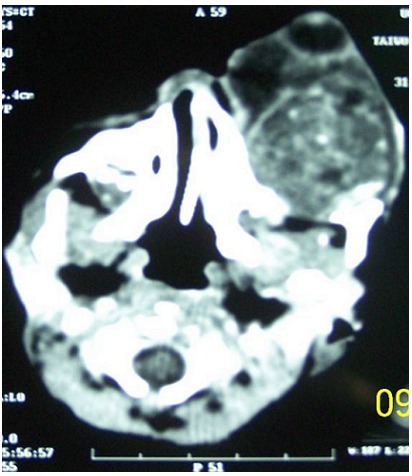
Computerised tomography showing the well circumscribed orbital tumour of mixed density with marked anterior displacement of the globe

We report the case of a female neonate who presented on the 2nd day of birth with a rapidly progressive left eye swelling from birth. Details of the pregnancy, birth and family history were unremarkable. The swelling rapidly filled the orbit and extruded the eye globe within 6 days of birth (Figure 1). Computerised tomography scan revealed a well circumscribed left retroocular mass of mixed density with splaying of the orbital walls and with increased vascularity (Figure 2) and which did not show any intracranial extension. A congenital orbital neoplasm was the working clinical diagnosis because of the rapid growth. The patient had a left modified exenteration on the 10^th^ day of life. The distorted globe and a well encapsulated orbital tumour were completely removed. Post operatively, the empty orbit was managed with daily saline, honey and sulfratulle dressing.

**Figure 3:**
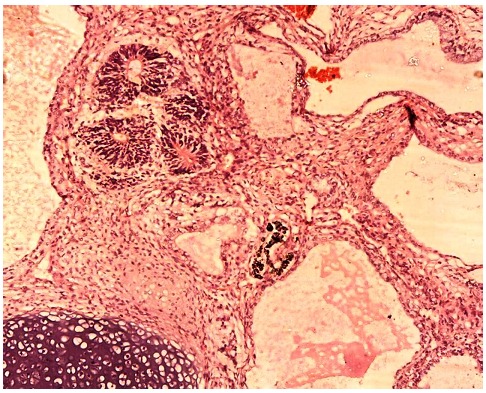
Haematoxylin & eosin stained sections (x40): showing neuroepithelium, cartilage and cystic spaces

On histopathological examination, the gross specimen measured 7x5x4cm in size. The cut sections showed that the eyeball, though encased by the tumour, was grossly delineated from it. The mass was well encapsulated. It had a predominantly greyish solid surface with multifocal micro- and macro-cystic spaces which were divided into nodules by prominent fibrous septa. Light microscopic examination revealed cysts lined predominantly by mature and immature squamous type, respiratory and colonic epithelium. The solid areas consisted of primitive neuroepithelial cells which comprised about 5% of the tumour and were forming tubules (Figure 3). The presence of primitive neuro-epithelium normally defines a teratoma as being immature. There was minimal mitotic activity observed. The solid areas also showed haphazard arrangement of different tissues which consisted of neuroglial, mature and immature cartilage, bone, hepatic tissue, pancreas, sheets of adipocytes, thyroid tissue, and primitive mesenchyme. The margins of resection were completely free of tumour. It was concluded that this was a Grade 1 teratoma (immature, probably benign) based on the grading system earlier described by Gonzalez-Crussi for extragonadal teratomas [10].

The patient was discharged on the 12th post-operative day with a clean, dry socket and was to be followed-up in the outpatient department in 2 weeks. At the follow-up visit, she was to be reviewed and fitted with an ocular prosthesis but she defaulted.

**Figure 4:**
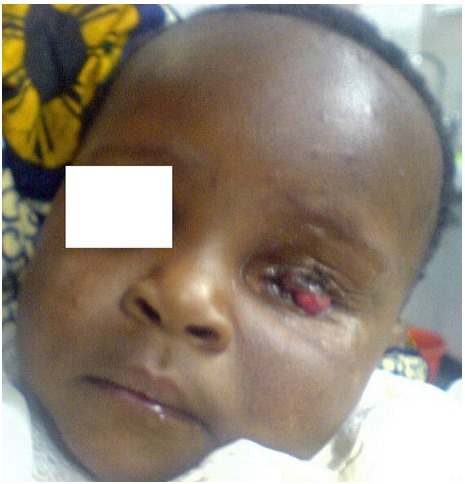
Contracted socket with lid notching and conjunctival exposure in same patient at six months of age having defaulted from follow-up

Contact with the patient was only re-established through the help of social health department of the hospital at 6 months of age (Figure 4) by which time, the left socket was already contracting and it was impossible to fit even our smallest prosthesis. The child was otherwise healthy and there was no local recurrence. The patient was referred for orbital plastic surgery to deepen the socket with the aim of inserting a large prosthesis to stimulate orbital bone development. However, despite extensive counselling, the patient's mother defaulted again. Through the concerted effort of the social health department, contact was again re-established at 24 months of age. The child was without recurrence. The socket at this time was severely contracted. The patient's mother was again counselled on the cosmetic and psychological implications of the poor bone development on that half of the face. The mother alluded to the fact that poor finance was the reason of the default. Also extensive plan for management was discussed with her. She was to be seen in a week for follow up but she defaulted again and has completely been lost to follow-up.

## Discussion

Orbital teratomas are exceedingly rare tumours which may occur at any age but present most often at birth with progressive, often alarming, unilateral proptosis.

Ameh et al in 1999 reported the first case in Nigeria from Zaria in a 1-day old female neonate [6]. That patient had a modified left exenteration and histopathological findings were similar to ours. Subsequently two other cases of orbital teratoma have been reported in Nigeria till date from Jos and Onitsha in the years 2000 and 2010 respectively [7, 8].

Prompt management of a congenital orbital teratoma is often rewarding. The tumour can usually be excised completely without the need for disfiguring surgeries like modified or radical exenteration. The retention or restoration of useful vision in the affected eye has been reported [4]. Where exenteration has been necessitated by severe proptosis and irreversible damage to the globe, early oculoplastic intervention can usually achieve favourable cosmetic outcomes. The major challenge in such cases is the need for revision surgery to maintain a balanced hemifacial and orbital development following the lack of local stimulation in the anophthalmic socket [3]. Intracranial extension of the tumour may occur and this may present a challenge in the surgical management.

A few reports have suggested that very rarely, a benign teratoma may recur as a malignant germ-cell tumour [2]. Garden and McManis described the recurrence as a malignant germ cell tumour in the orbit of a child three years after an apparently complete excision of an orbital-intracranial teratoma with preservation of the globe [2]. Although there is the possibility that the malignant germ cell tumour arose de novo in that case, the long term malignant potential of apparently benign teratomas of the orbit is inferred.

Though, this is the fourth documented case that has ever been described in our country, it is the first from South-western Nigeria and adds to the growing literature of this rare tumour. The patient's parents in our case were semi-literate and every attempt was made through direct counselling by the medical team and home visitation on the part of the social health workers, to communicate to them; our management goals and prognosis for the patient. After 24 months of follow up the patient could not be contacted again through the address we had because her family had move residence.

## Conclusion

In conclusion, congenital immature orbital teratoma is potentially curable; however, in this case we note that in spite of early multidisciplinary intervention, a good cosmetic outcome was not achieved due to interplay of contemporaneous socioeconomic factors as highlighted.
